# The mobility of small vacancy/helium complexes in tungsten and its impact on retention in fusion-relevant conditions

**DOI:** 10.1038/s41598-017-02428-2

**Published:** 2017-05-30

**Authors:** Danny Perez, Luis Sandoval, Sophie Blondel, Brian D. Wirth, Blas P. Uberuaga, Arthur F. Voter

**Affiliations:** 1 Theoretical Division T-1, Los Alamos National Laboratory, Los Alamos, New Mexico 87545 USA; 2 Materials Science and Technology MST-8, Los Alamos National Laboratory, Los Alamos, New Mexico 87545 USA; 3 Department of Nuclear Engineering, University of Tennessee, Knoxville, TN 37996 USA; 4 AMA Inc., Thermal Protection Materials Branch, NASA Ames Research Center, Moffett Field, California 94035 USA

## Abstract

Tungsten is a promising plasma facing material for fusion reactors. Despite many favorable properties, helium ions incoming from the plasma are known to dramatically affect the microstructure of tungsten, leading to bubble growth, blistering, and/or to the formation of fuzz. In order to develop mitigation strategies, it is essential to understand the atomistic processes that lead to bubble formation and subsequent microstructural changes. In this work, we use large-scale Accelerated Molecular Dynamics simulations to investigate small (*N* = 1,2) V_*N*_He_*M*_ vacancy/helium complexes, which serve as the nuclei for larger helium bubble growth, over timescales reaching into the milliseconds under conditions typical of the operation of fusion reactors. These complexes can interconvert between different I_*L*_V_*N*+*L*_He_*M*_ variants via Frenkel pair nucleation (leading to the creation of a additional vacancy/interstitial pair) and annihilation events; sequences of these events can lead to net migration of these embryonic bubbles. The competition between nucleation and annihilation produces a very complex dependence of the diffusivity on the number of heliums. Finally, through cluster dynamics simulations, we show that diffusion of these complexes provides an efficient pathway for helium release at fluxes expected in fusion reactors, and hence that accounting for the mobility of these complexes is crucial.

## Introduction

Nuclear fusion is a promising carbon-neutral source of energy. However, practical schemes for fusion energy production, such as magnetic confinement, place extremely stringent demands on materials, in terms of heat and particle fluxes. At first sight, tungsten appears to be an ideal first wall candidate, due to its very high melting point and low sputtering yield. However, exposure to the fusion plasma, and in particular to the helium generated by the fusion reactions, has proven to be extremely detrimental, causing drastic changes in surface microstructure such as blistering^1^ or the formation of fuzz^2–5^, which can be described as a “forest” of nanowires that appear to grow on the surface of the material. Such surface modifications can negatively affect the performance of the material. Due to the immense technological importance of the problem, the need for fundamental insight into the mechanisms that drive microstructural evolution has motivated a wide range of computational studies of He in tungsten, including the properties of interstitial He clusters^6–8^ and their interactions with various types of defects^9–15^, the conversion of He interstitial clusters into nanobubbles^8, 16, 17^, and their subsequent growth through absorption of additional He atoms^18–22^.

In this paper, we focus on the diffusive behavior of the nuclei of He bubbles: small vacancy/helium (V_*N*_He_*M*_) complexes. These complexes can form spontaneously following self-trapping of interstitial He clusters (the nucleation of a Frenkel pair induced by a cluster of interstitial He atoms, which is prevented from recombining by He atoms filling the vacancy^18, 23, 24^), or by interstitial He atoms binding with preexisting vacancies (formed either thermally, due to processing, or from radiation damage). One crucial question that needs to be addressed to enable accurate prediction of He-driven microstructure evolution is whether these complexes can diffuse, either randomly or under the influence of external stress or temperature gradients. In current fusion models, these are usually assumed either to be immobile^25, 26^, or to diffuse through a conventional surface-mediated mechanism whereby vacancies exchange positions with neighboring matrix atoms^27^. However, Gao *et al*.^28^ have recently observed that analogous complexes in Fe can diffuse by sequences of emission and annihilation of Frenkel pairs. In the following, we revisit this problem for He in W and characterize the dependence of the mobility on the He and vacancy content of these defects.

Note that these complexes are also relevant to nuclear fission applications where helium is a common fission or transmutation product. As such, their energetics and thermodynamics have been extensively investigated for a range of different metals, see e.g., refs 29 and 30. However, given the complex nature of these defects, exhaustively identifying the relevant kinetics mechanisms and estimating the corresponding transition rates is difficult; direct, MD-based, simulation approaches are therefore extremely desirable in order to investigate their dynamics. In this work, Accelerated Molecular Dynamics (AMD)^31^ simulations using the recently-introduced Parallel Trajectory Splicing (ParSplice) method^32^ deployed on up to 100,000 cores on the Trinity supercomputer in Los Alamos National Laboratory have been used to to reach milliseconds of simulation time. As detailed in the Methods section, ParSplice is a technique to parallelize the generation of long atomistic trajectories in the time domain, leveraging parallel computers to reach very long timescales on relatively small systems. As shown below, this allows for the direct observation of the motion of the complexes and for the characterization of their diffusivity. Through a mesoscale cluster-dynamics model, we show that accounting for the mobility of these complexes is critical to the the accurate prediction of the evolution of the first wall in fusion condition.

## Results

**Figure 1:**
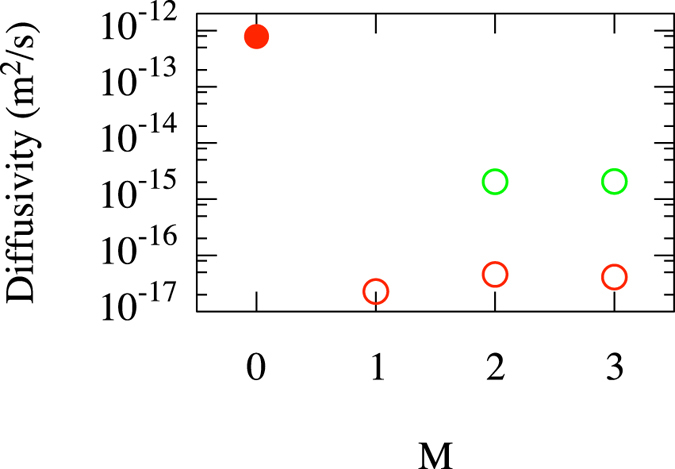
Diffusivity of low helium content V_*N*_He_*M*_ complexes at *T* = 1000 *K*. Red: *N* = 1; Green: *N* = 2. Filled symbols correspond to estimates of the diffusivity, while open symbols are upper bounds at a 90% confidence level for cases where the complexes did not diffuse on accessible simulation timescales. The filled circle point represents a tungsten mono-vacancy containing no He.

A first regime of interest is at small helium content (0 ≤ *M* ≤ 3), which is relevant to understanding the effect of He exposure on materials containing pre-existing vacancies. Indeed, such complexes would not spontaneously form in bulk tungsten, as around 6 or 7 He interstitials appear to be required to trigger a spontaneous trap mutation (or self-trapping) event^8, 16^. Note however that the trap mutation process has been observed to occur at smaller M near surfaces^10, 13^. As shown in Fig. 1, the diffusivity of a bare vacancy (*N* = 1, *M* = 0) in bulk tungsten is ~10^−12^ m^2^/s at 1000 K. The addition of a single He atom however completely immobilizes the complex on ms timescales, which implies a diffusivity below 10^−16^ m^2^/s with 90% confidence. The vacancy remains immobile on similar timescales for *M* = 2 and 3. The case of the di-vacancy (*N* = 2) is slightly more complex: as it is only weakly bound, the dimer readily breaks and reforms; over long times, we expect the dimer to break apart and the individual vacancies to diffuse freely. The addition of a single He leads to the immobilization of only one of the vacancies, while the other remains free to diffuse. When the dimer temporarily reforms, the He atom can hop from one vacancy to the other, freeing one and immobilizing the other. This corresponds to the well known vacancy transport mechanism for helium that is mediated by transient formation of vacancy-rich complexes^33^. The addition of a second He atom again completely immobilizes the pair over timescales of tens of *μ*s, leading to an upper bound on the diffusivity of around 10^−15^ m^2^/s. As before, an additional He renders the complex immobile over the timescales simulated here.

While a single He per vacancy appears sufficient to immobilize the complexes over ms timescales, this does not exclude the possibility of rarer mechanisms occurring on even longer timescales. For example, He atoms could hop out of the vacancy, setting it free until an He is recaptured^34^. Given the strong binding of He to vacancies (dissociation energies in the range 2.9–4.5 eV have been reported for single He atoms leaving V_2_He_*M*_ complexes with 1 ≤ *M* ≤ 5^35^, in agreement with density functional theory (DFT) calculations)^17^ the timescales over which such process occurs is however out of reach of the current simulations.

Increasing *M* further dramatically changes the behavior of these complexes. It is indeed well known that a large *M*-to-*N* ratio leads to bubble growth whereby tungsten Frenkel pair nucleation progressively increases the size of the bubble, as the He prevents recombination of tungsten defects by expanding to fill the newly created vacancy. As this process repeats following the absorption of additional He atoms, the excess interstitials accumulate around the bubble. These are eventually released, often in the form of small prismatic dislocation loops, a process referred to as loop punching^22, 36–40^.

**Figure 2:**
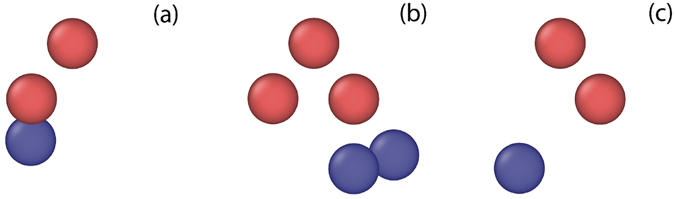
Illustration of a typical nucleation/annihilation sequence for *N* = 1 and *M* = 15. Red and blue spheres represent tungsten vacancies and interstitials, respectively. He atoms and bulk tungsten atoms are not shown, for clarity. Panel (a) I_1_V_2_He_15_ variant; (**b**) following a Frenkel pair nucleation event, the system transforms into a I_2_V_3_He_15_ variant; (**c**) the system reverts to a I_1_V_2_He_15_ variant following a Frenkel pair annihilation event. The net result of this sequence of events is a shift in the center-of-mass position of the cluster. The system is viewed along a [110] direction in all frames.

**Figure 3:**
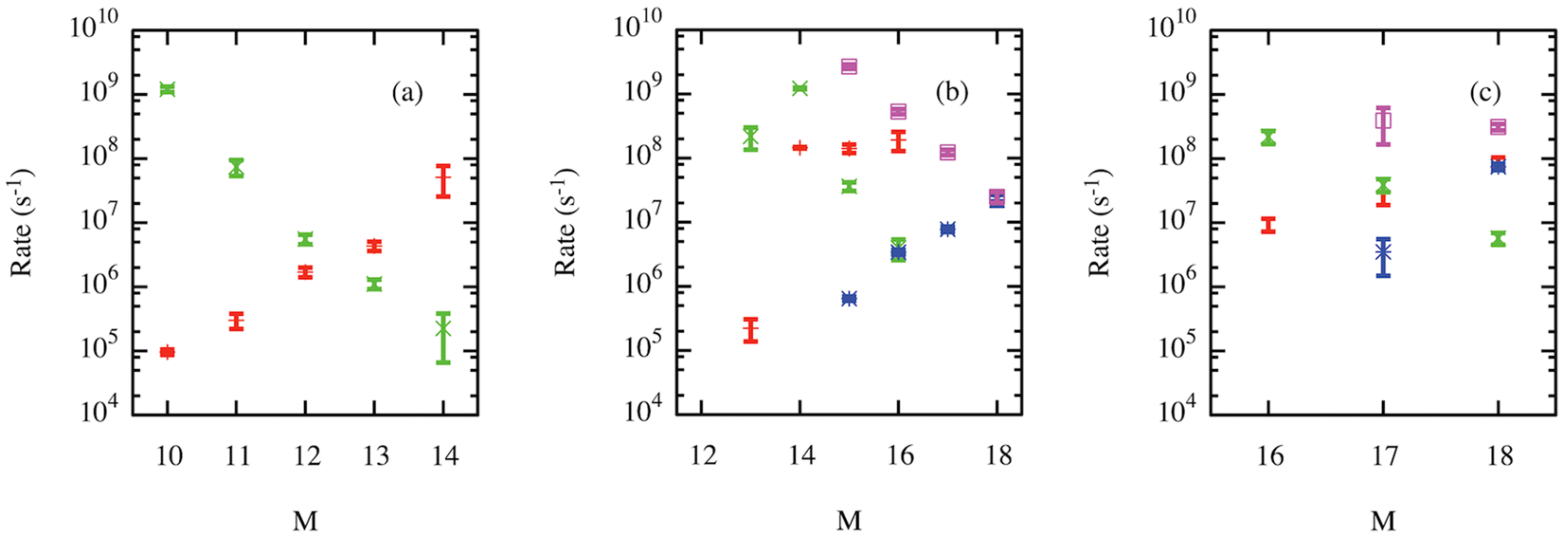
Frenkel pair nucleation and annihilation rates at *T* = 1000 *K* as a function of *M*. Red crosses: nucleation rate for *N* = 1; Green x: annihilation rate for *N* = 1; Blue triangles: nucleation rate for *N* = 2; Magenta squares: annihilation rate for *N* = 2. (**a**) transition rates between *N* + *L* = 1 and 2; (**b**) transition rates between *N* + *L* = 2 and 3; (**c**) transition rates between *N* + *L* = 3 and 4.

A fact that has perhaps been less appreciated is that this Frenkel pair nucleation process is reversible. For example, “untrapping” through absorption of a self interstitial has been observed at low *M* in iron^41^ and in tungsten^8^; this reaction annihilates a vacancy and returns the He atoms to interstitial positions. A corresponding sequence of nucleation/annihilation for the complexes is shown in Fig. 2. Through these competing mechanisms, V_*N*_He_*M*_ complexes can be found in a range of variants I_*L*_V_*N*+*L*_He_*M*_ that result from *L* cumulative Frenkel pair nucleation events. Note that, in contrast to the “untrapping” reaction, at least one vacancy always remains due to the stoichiometry of the considered complexes, so that interstitial He are never emitted. From the ParSplice simulations, we extracted the nucleation and annihilation rates for interconversion between the most likely variants; these are reported in Fig. 3. The rates at which nucleation and annihilation occur prove very sensitive to helium content, but in opposite ways: as *M* increases, the nucleation rate increases, while the annihilation rate decreases. For example, rates for V_1_He_*M*_ ↔ I_1_V_2_He_*M*_ reactions (c.f. panel (a) of Fig. 3) depend exponentially on the helium content *M*: for *M* = 10, nucleation requires on the order of 10 *μ*s, but subsequent annihilation less than 1 ns, while the roles are reversed for *M* = 14.

This is of course related to the fact that He pressure favors nucleation, but counteracts annihilation. Note that the number *L* of interstitials that are already present in the variant affects the rates of subsequent nucleations and annihilation: as a rule of thumb, interstitials favor additional nucleation but inhibit annihilation. This is consistent with the fact that self interstitials bind together rather strongly: DFT predicts a binding energy of around 2.5 eV for an interstitial pair in bulk^17^. The energy barrier for nucleation is thus expected to decrease due to the presence of other interstitials, while that of annihilation will increase, as it entails “unbinding” an interstitial. This is consistent with the tendency of Frenkel pair nucleations to occur in the vicinity of pre-existing interstitials that was previously observed in long-timescale simulations of bubble growth^22^. Analysis of the current simulations further shows that nucleation and annihilation tend to occur in a way that leaves the vacancy clusters as compact as possible, i.e., so as to maximize the number of vacancy first neighbors. A similar behavior was recently observed in AMD simulations of trap mutation in the vicinity of a bubble; in this case, vacancies located at corners of the bubble were the primary targets for annihilation^15^.

**Figure 4:**
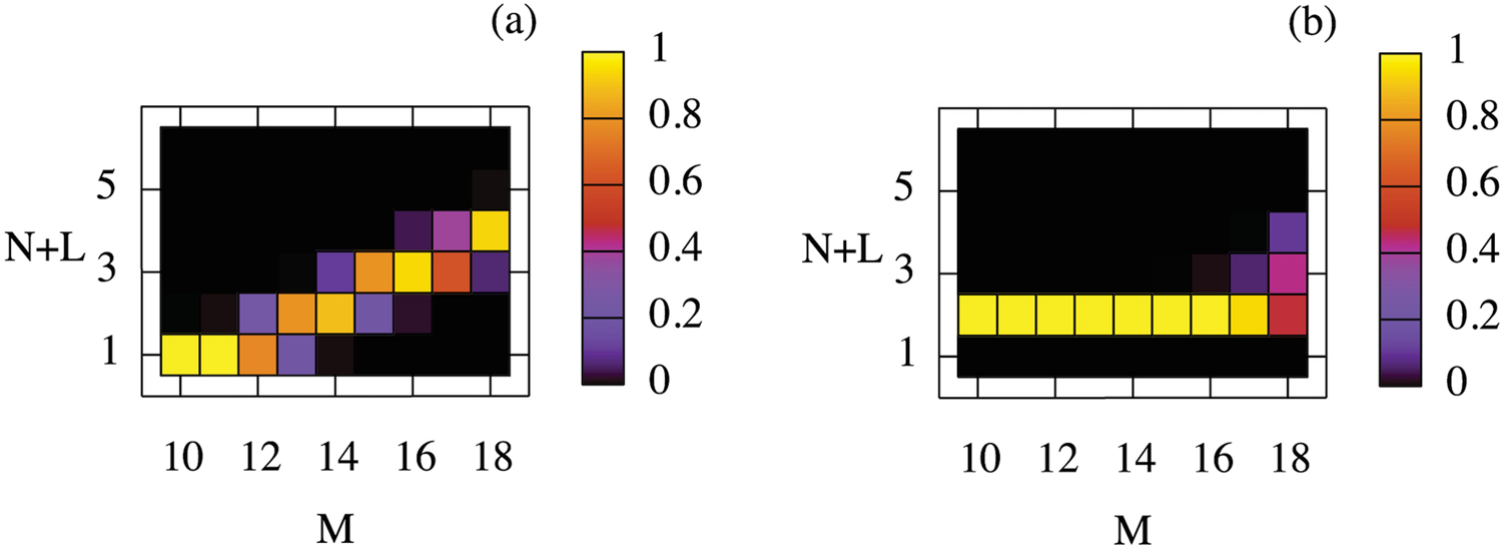
Equilibrium probabilities of observing different *M*, *N* + *L* variants at given values of the helium content *M* at *T* = 1000 *K*. Probabilities are normalized so that ${\sum }_{L}{p}_{M,N+L}=1$. (**a**) *N* = 1; (**b**) *N* = 2.

Assuming that these processes are the only ones that operate, one can infer the equilibrium distribution of the different variants by solving for the steady state of the corresponding rate equations. The results are shown in Fig. 4, where the color at a given *M*, *N* + *L* coordinate encodes the probability of observing that specific variant at the corresponding value of the helium content *M*. These probabilities are normalized so that ${\sum }_{L}{p}_{M,N+L}=1$. The results show that the equilibrium value of *L* increases with increasing *M*. This is expected, given the sharp increase in nucleation rates with increasing *M*. For *N* = 1, multiple variants start to coexist around *M* = 12 (two or more values of *N* + *L* have significant occurrence probabilities). Coexistence is usually limited to 2 variants, but in some cases, up to 3 can coexist. Comparison of the *N* = 1 and *N* = 2 cases again points to the importance of interstitial binding: without an extra interstitial to promote further nucleation, variants with 3 vacancies only become common by *M* = 17 for *N* = 2, while only *M* = 14 is required for *N* = 1.

**Figure 5:**
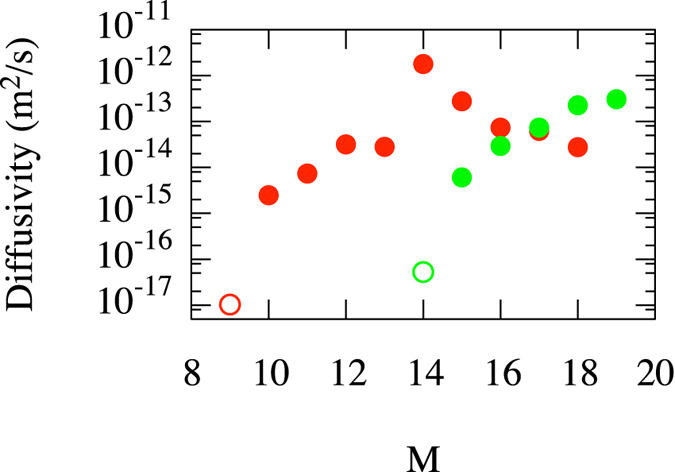
Diffusivity high helium content V_*N*_He_*M*_ complexes at *T* = 1000 *K* as estimated from kinetic Monte Carlo simulations informed by ParSplice simulations. Red: *N* = 1; Green: *N* = 2. Filled symbols correspond to estimates of the diffusivity, while open symbols are upper bounds at a 90% confidence level for cases where the complexes did not diffuse on accessible simulation timescales.

This interconversion process between variants is key to confer mobility to these complexes. Indeed, annihilation is not restricted to the reverse process of the last nucleation: as interstitials can typically easily diffuse around the vacancies, any one of those vacancies can in principle be annihilated. As shown in Fig. 2, this eventuality gives rise to net motion of the cluster through sequential nucleation/interstitial migration/annihilation events. This process has recently been observed in AMD simulations of He in Fe by Gao *et al*.^28^, but they did not characterized the *N*,*M* dependence of the mobility of these defects. Indeed, for such a mechanism to give rise to high mobility, *both* nucleation and annihilation rates need to be high; otherwise, the mobility will be limited by the slowest process. Due to the strong dependence of the nucleation/annihilation rates on *M*, and the contribution from different variants to mobility, one can expect the mobility to strongly depend on *M*. To avoid any biases due to limited direct simulation times, the diffusivity of clusters as a function of *M* was estimated through a kinetic Monte Carlo model parameterized using the rates shown in Fig. 3. To be consistent with observations, vacancy nucleation and annihilation only occurs at sites that maximize the compactness of the resulting complexes, i.e., so that the number of first-neighbor vacancy pairs is maximized. The results, reported in Fig. 5, show that the diffusivity is indeed very sensitive to the helium content. For example, for *N* = 1, Frenkel pair nucleation was not observed on ms timescales in bulk tungsten for *M* = 9, which implies a diffusivity below about 10^−17^ m^2^/s at 1000 K. The addition of a single He leads to a dramatic increase in diffusivity up to ~10^−15^ m^2^/s. In this case, mobility is driven by V_1_He_*M*_ ↔ I_1_V_2_He_*M*_ reactions. From Fig. 3, it is apparent that mobility is initially limited by the nucleation rate. However, by *M* = 12,13 the two rates cross-over and annihilation becomes the rate-limiting step, hence the slight decrease in diffusivity. By *M* = 14, the diffusivity suddenly increases by 2 orders of magnitude, reaching ~10^−12^ m^2^/s. This corresponds to the point where the dominant reaction becomes I_1_V_2_He_*M*_ ↔ I_2_V_3_He_*M*_ (c.f. Fig. 4). The cross-over from nucleation-limited to annihilation-limited behavior occurs between *M* = 14 and 15, which explains the lower diffusivity at *M* = 15. For *M* = 16, three variants (with 1, 2, and 3 vacancies) actually contribute to the mobility. At *M* = 17, the dominant reaction becomes I_2_V_3_He_*M*_ ↔ I_3_V_4_He_*M*_, which also corresponds to the cross-over point for that reaction.

Due to the combined effects of the change in the equilibrium distribution of the different variants and of the corresponding nucleation and annihilation rates, the dependence of the diffusivity on *M* across the *N* = 1 series is very complex, with a very low mobility below *M* = 10, a strong peak at *M* = 14 and a subsequent decrease at larger *M*. Over the same range of *M*, the behavior of the *N* = 2 series is comparatively much simpler: except for *M* = 18, the dominant reaction is V_2_He_*M*_ ↔ I_1_V_3_He_*M*_, whose cross-over point is at *M* = 18. The diffusivity is thus monotonic over the probed range of *M*. Again, a sufficiently large *M* (>14) is required for the complexes to move; they are otherwise immobile on the ms timescales accessible here. Note that at their peak mobility, the complexes can diffuse roughly as fast as a bare vacancy, but that a typical complex often diffuses much slower. While these complexes diffuse extremely slowly compared to interstitial He clusters (~10^−9^ m^2^/s at 1000 K)^8^, this is nonetheless sufficient to significantly affect the overall evolution of He content in the material in conditions relevant to fusion applications, as will be shown below.

Comparison of the *N* = 1 and *N* = 2 complexes also shows that absorption or emission of interstitials can have a significant impact on the diffusivity. For example, the transition I_1_V_2_He_14_ → V_2_He_14_ + SIA leads to a dramatic drop in the diffusivity by many orders of magnitude because the bound interstitial was essential to promote the nucleation of an additional Frenkel pair required for the cluster to move. However, losing an interstitial can also lead to an increase in diffusivity when the mobility is instead limited by annihilation, as in I_1_V_2_He_18_ → V_2_He_18_ + SIA. While emission of interstitials (complete break-away from the bubble) was not observed over the timescale accessible here, it is well established that He bubbles emit interstitials during their growth: recent estimates based on density functional theory yield binding energies that range between 2 and 3 eV^16^. As the corresponding emission barriers are even higher, such events would require on the order of seconds (or even more) to occur, a time that is long compared to the timescales accessed here, but short enough to be relevant to reactor operation. Similarly, absorption of tungsten interstitials can also occur, especially under irradiation conditions. Our results show that such interactions will affect the mobility of these complexes.

## Discussion

In order to assess the importance of this mechanism to describe the evolution of the tungsten first wall in fusion conditions, we compare the outcome of mesoscale cluster dynamics simulations when the complexes are considered to be mobile and immobile. These simulations are carried out with the Xolotl model of microstructural evolution^11^. Xolotl is a recently developed spatially-dependent cluster dynamics code solving the drift-diffusion-reaction equations for the evolution of the population of different defects induced by He exposure in fusion conditions. Present calculations were carried out in a 1D geometry using a refined mesh spacing near the surface. Briefly, introduction of He in the wall follows a flux profile (flux of He atoms vs depth) corresponding to He atoms incoming with an energy of 100 eV, as determined by direct molecular dynamics simulations^11^. These then diffuse through the material, forming increasingly large interstitial clusters as they collide. Once these interstitial clusters reach a large enough size, they spontaneously trap mutate, leading to the formation of V/He complexes. The thermodynamics and kinetics of interstitial clusters — including diffusivities, breakup, and trap mutation rates — appropriately modified to account for the proximity of free surfaces^11^, have been parameterized from extensive atomistic simulation^8, 11^; the reader is refered to these publications for complete details. These V/He complexes grow into full-fledged He bubbles through the capture of additional interstitial helium (either as single atoms or as clusters) and the emission of W interstitials through Frenkel pair nucleation in order to maintain the number of He per vacancy at 4. Calculations are here initialized from a pristine W wall, i.e., no vacancies or interstitials (either W or He) are initially present; therefore, all V/He complexes are assumed to form following the trap mutation of interstitial He complexes.

**Figure 6:**
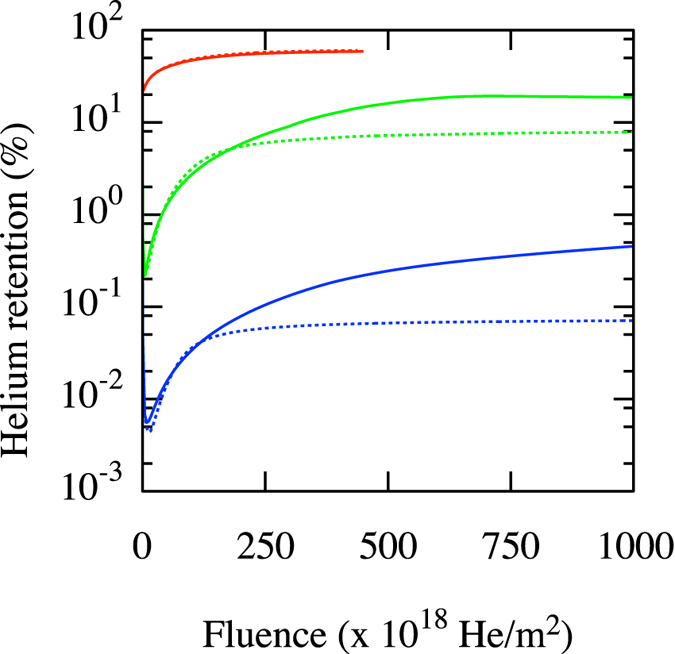
Helium retention as a function of fluence. Red: 4 × 10^25^ He/m^2^/s; Green: 4 × 10^23^ He/m^2^/s; Blue: 4 × 10^22^ He/m^2^/s. Continuous and dashed lines correspond to immobile and mobile complexes, respectively.

**Figure 7:**
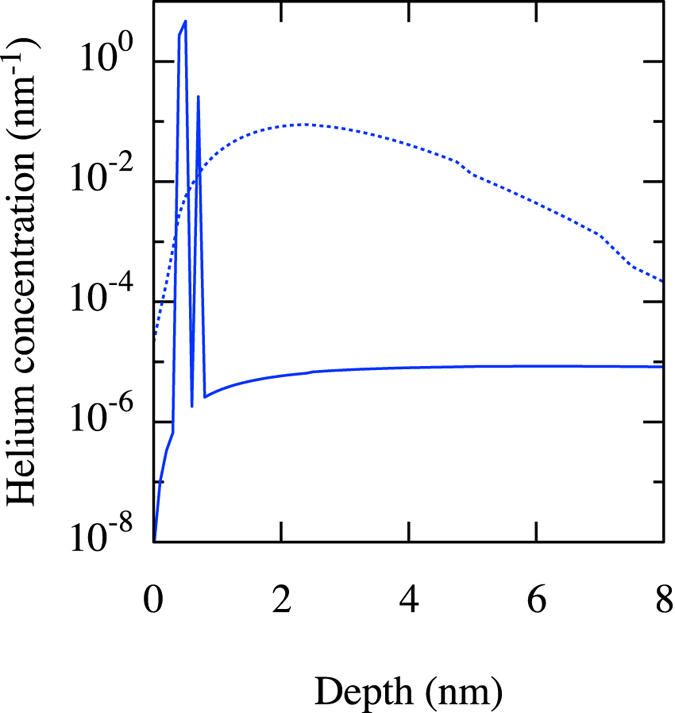
Helium concentration as a function of depth for a flux of 4 × 10^22^ He/m^2^/s at a fluence of 4 × 10^20^ He/m^2^. Continuous and dashed lines correspond to immobile and mobile complexes, respectively.

Given that Xolotl does not resolve the number of interstitials attached to He/V complexes, we use effective diffusion rates based on the AMD results given above, i.e., 10^−14^ m^2^/s for V_1_He_*M*_ complexes, 10^−13^ m^2^/s for V_2_He_*M*_ and V_3_He_*M*_ complexes. For simplicity, larger clusters are considered to be immobile. We consider 3 He fluxes: 4 × 10^25^, 4 × 10^23^ and 4 × 10^22^ He/m^2^/s, the last two of which are typical of what is expected on the divertor plate in ITER^42^. As shown in Figs 6 and 7, both the overall retention of He within the tungsten wall and the distribution of He atoms within the sample are significantly affected by taking the mobility of the small V/He complexes into account. Specifically, the amount of He that remains within the sample decreases and their distribution shifts deeper into the sample. This is due to the fact that trap mutation tends to occur relatively close to the surface, where the concentration of He is high and where the presence of the surface itself facilitates the process. If these complexes are mobile, they can diffuse and reach the surface (which acts as a sink), or diffuse deeper into the bulk, as compared to the case where they are considered to be immobile. This extra channel for He release becomes extremely efficient at lower fluxes, leading to a significant (up to five-fold) reduction in He retention at low fluxes (c.f. Fig. 6). Note that the effect is more pronounced at lower fluxes, since the complexes then have more time to diffuse until they grow to a point where they become immobile; for fluxes higher than 4 × 10^25^ He/m^2^/s, the effect of complex diffusion is negligible.

Thus, when small He/V clusters are mobile, they have a huge impact on He retention. If even larger clusters (>3 vacancies) were also considered to be mobile, the impact would be even greater. These results therefore demonstrate the crucial importance of accounting for the mobility of these complexes in order to adequately predict the evolution of He in the tungsten wall in fusion conditions; and just as important, these results demonstrate that incorrect results can be inferred from only performing molecular dynamics simulations to investigate helium behavior resulting from implantation that is many of orders of magnitude accelerated compared to experimental conditions.

Finally, while our study is limited to small complexes, the results suggest that a similar mechanism could allow somewhat larger bubbles to move. Motion of bubbles, e.g., in response to elastic stresses created by nearby features such as surfaces and grain boundaries, could significantly affect microstructure evolution. Current models usually assume that bubbles are immobile^25, 26^ or that they diffuse through the same (internal) surface-mediated diffusion process as pores^27^. Internal surface diffusion was observed in AMD simulations of bubble growth, but the available timescales were insufficient for the bubble to move significantly. It is also possible for bubbles to move by matrix atoms diffusing from one side of the bubble to the other through the gas phase interior of the bubble (vapor-mediated mechanism)^43, 44^, or by emission and reabsorption of vacancies (volume-mediated mechanism)^43, 44^. Our results suggest a different kind of volume-based mechanism, one that is mediated by interstitials instead of vacancies through Frenkel pair nucleation and annihilation. While this process has not been reported in MD or AMD simulations of bubble growth, this is probably a consequence of the rather fast growth rates employed in those simulations. In order for the nucleation/annihilation mechanism to be effective, annihilation has to occur sufficiently quickly on the timescale of additional He arrival, otherwise it will be gradually suppressed by the increasing He pressure. Based on recent AMD simulations, intake rates down to 10^8^ He/s^22^ appear to still be in the “irreversible” growth regime where annihilation is unlikely. However, the order of magnitude of the nucleation/annihilation rates observed here (c.f. Fig. 3) suggests that growth rates on the order of 10^6^ or 10^7^ He/s could be in a regime where both processes would be active. The efficiency of this mechanism for larger bubbles is under investigation.

## Methods

In order to access timescales that are sufficient to enable the motion of V_*N*_He_*M*_ complexes in conditions typical of the first wall in fusion reactors (*T* ~ 1000 *K*), we use Parallel Trajectory Splicing (ParSplice)^45^, a recently introduced AMD method that improves upon its predecessor, Parallel Replica Dynamics^32, 46^. AMD techniques are designed to extend the simulation timescales of systems evolving through rare, activated, events between persistent states. ParSplice is based on the concept that, for such systems, a state-to-state trajectory can be assembled from independently generated trajectory segments provided that the system has remained for a correlation time *τ*_*c*_ within a state *prior to the start* of the segment and has remained within a (possibly different) state for *τ*_*c*_ *prior to the end* of the segment. One can show that the error introduced by this decomposition into independent segments scales as exp(−*Cτ*_*c*_), where *C* is a positive constant that depends on the separation of timescales between intra-state relaxation and escape. As segments are independent from one another, they can be concurrently generated on a parallel computer and subsequently assembled into a long state-to-state trajectory. This allows for a *time*-*wise* parallelization strategy that is extremely powerful for small systems, as it enables the extension of the simulation timescales by a factor of *O*(*N*_parallel_), where *N*_parallel_ is the number of parallel MD instances. A key innovation of ParSplice is that segments can be concurrently generated in multiple states, according to the expected likelihood that they will be used in the construction of the trajectory. Details can be found in the original publication^45^.

One of the powerful features of ParSplice is that states can be arbitrarily defined. This freedom can be exploited so as to maximize the separation of timescales between relaxation within states and escape from states (and hence maximize *C*). In the current case, helium atoms tend to reorganize very quickly within the vacancies compared to the timescale for transitions involving tungsten atoms, the events that dictate migration of the cluster itself, occur; states are therefore defined based on the topology of the tungsten atoms alone.

Calculations were carried out using an embedded atom method (EAM) description of the interatomic interactions, with W-W interactions from Ackland and Thetford^47^ and modified by Juslin and Wirth^48^, He-He interactions from Beck^49, 50^, modified by Morishita *et al*.^51^, and He-W interactions from Juslin and Wirth^48^. V_*N*_He_*M*_ (*N* = 1,2; 0 ≤ *M* ≤ 19) complexes are created in a cubic simulation cell containing 8 × 8 × 8 bcc tungsten unit cells at the bulk lattice constant appropriate for the simulation temperature of 1000 *K*. Temperature is controlled through a Langevin thermostat with a friction of 10^12^ s^−1^. *N*_parallel_ ranged between 2,400 and 200,000 depending on the rate at which the complexes evolve. With this simulation setup, ParSplice enables high-fidelity simulations over timescales ranging from *μ*s to ms.

## Conclusion

The behavior of small V_*N*_He_*M*_ complexes in tungsten in conditions relevant to nuclear fusion was investigated using AMD simulations over timescales reaching into the milliseconds. The simulations show that these complexes can interconvert between different I_*L*_V_*N*+*L*_He_*M*_ variants through Frenkel pair nucleation and annihilation events. Sequences of nucleation/annihilation allow these defects to diffuse. The competing kinetics of these two processes lead to a complex dependence of the diffusivity on the number of helium atoms within the bubble and on the number of interstitials that are bound to the complex. At their peak mobility, the diffusivity of these complexes is similar to that of a bare vacancy. Cluster dynamics simulations of material evolution in fusion-relevant conditions show that the diffusion of these clusters is an efficient channel for He to leave the material, and that it modifies the depth distribution of larger helium bubble complexes, highlighting the importance of considering this process in predictions of microstructural evolution.
